# Sphingolipid Metabolism: New Insight into Ceramide-Induced Lipotoxicity in Muscle Cells

**DOI:** 10.3390/ijms20030479

**Published:** 2019-01-23

**Authors:** Cécile L. Bandet, Sophie Tan-Chen, Olivier Bourron, Hervé Le Stunff, Eric Hajduch

**Affiliations:** 1 INSERM UMRS 1138, Sorbonne Université; Sorbonne Paris Cité, Université Paris Descartes, Université Paris Diderot; Centre de Recherche des Cordeliers, 75006 Paris, France; cecile.bandet@gmail.com (C.L.B.); sophie.tan@crc.jussieu.fr (S.T.-C.); olivier.bourron@aphp.fr (O.B.); 2 Institut Hospitalo-Universitaire ICAN, 75013 Paris, France; 3 Sorbonne Université, Assistance Publique-Hôpitaux de Paris, Service de Diabétologie et Maladies Métaboliques, Hôpital Pitié-Salpêtrière, 75013 Paris, France; 4 CNRS UMR 9198 Institut des Neurosciences Paris Saclay (Neuro-PSI), Université Paris-Saclay, 91400 Orsay, France; herve.le-stunff@paris7.jussieu.fr

**Keywords:** sphingolipids, ceramide, insulin, diabetes, DAG

## Abstract

Insulin-resistance is a characteristic feature of type 2 diabetes (T2D) and plays a major role in the pathogenesis of this disease. Skeletal muscles are quantitatively the biggest glucose users in response to insulin and are considered as main targets in development of insulin-resistance. It is now clear that circulating fatty acids (FA), which are highly increased in T2D, play a major role in the development of muscle insulin-resistance. In healthy individuals, excess FA are stored as lipid droplets in adipocytes. In situations like obesity and T2D, FA from lipolysis and food are in excess and eventually accumulate in peripheral tissues. High plasma concentrations of FA are generally associated with increased risk of developing diabetes. Indeed, ectopic fat accumulation is associated with insulin-resistance; this is called lipotoxicity. However, FA themselves are not involved in insulin-resistance, but rather some of their metabolic derivatives, such as ceramides. Ceramides, which are synthetized de novo from saturated FA like palmitate, have been demonstrated to play a critical role in the deterioration of insulin sensitivity in muscle cells. This review describes the latest progress involving ceramides as major players in the development of muscle insulin-resistance through the targeting of selective actors of the insulin signaling pathway.

## 1. Introduction

A worldwide obesity and diabetes epidemic has been spreading in humans all over the world in the last four decades. According to the World Health Organization, in 2014, 422 million people had diabetes compared to 108 million in 1980. Estimates report that in 2045, the number of diabetics will reach at least 629 million [1]. Diabetes is a condition that is characterized by a chronic hyperglycemia. After several years of unbalanced diabetes, complications altering the quality of life of patients appear and can lead to premature death. These complications are classified into two groups: microvascular complications, such as retinopathy, neuropathy, and nephropathy; and macrovascular complications, such as stroke and myocardial infarction [2]. There are two main types of diabetes: (i) type 1 diabetes (T1D), resulting from a total insulin deficiency (insulinopenia) subsequent of destruction of insulin-producing cells, which accounts for 10% of diabetes cases; and (ii) type 2 diabetes (T2D), the most common diabetes (90% of diabetes cases), characterized by two major cell dysfunctions: insulin resistance of peripheral organs such as liver, adipose tissue, and skeletal muscles, and partial insulinopenia from the β-cells. Insulin resistance results in a failure of insulin-sensitive tissues to respond to the insulin signal. T2D is associated with many susceptibility genes whose expression depends on environmental factors, such as the level of physical activity, and both hyper-carbohydrate and high fat diets [3]. As soon as insulin resistance appears, the pancreas produces more insulin to maintain normoglycemia. However, when the insulin secretory function is insufficient to counterbalance increasing insulin resistance, hyperglycemia appears. This insulin secretion dysfunction is linked to genetic/epigenetic and environmental factors [4]. 

T2D is concomitant with alterations of carbohydrate/lipid metabolism, and particularly of dyslipidemia, which have major consequences for cardiovascular diseases and insulin resistance, a key actor of T2D. During obesity, ectopic fatty acids (FA) accumulated into non-adipose tissues are metabolized as sphingolipid derivatives, such as ceramides. Interestingly, ceramides are among the most active lipid second messengers to inhibit key proteins of the insulin signaling pathway and to induce β-cell apoptosis. Defining new therapeutic targets to treat these metabolic disorders and their subsequent complications is one of the major challenges for clinical research in the future. 

Although ceramide excess can affect all insulin-sensitive tissues, we will focus this review on the impact of these lipids on skeletal muscle, tissue that is responsible for 30% of basal glucose utilization, and from 70 to 90% in response to insulin in healthy individuals [5,6,7,8]. Indeed, skeletal muscles are major tissues for the maintenance of normoglycemia and are therefore a primary target for insulin resistance [9]. We will provide an overview of our present understanding of known machineries and mechanisms by which ceramides affect negatively glucose homeostasis in skeletal muscles. 

## 2. Type 2 Diabetes Pathophysiology: From Physiological Insulin Signaling to Energy Homeostasis Disruption

Insulin is an anabolic hormone which induces many metabolic pathways in its different target tissues, resulting in a hypoglycemic effect. These cellular responses are the result of the activation of a very specific cascade signaling pathway whose mechanisms are now well characterized.

Initially, insulin binds its receptor, which is localized in the plasma membrane (PM) of most cells, but much more so in “insulin-sensitive” cells (liver, adipose tissue, skeletal muscles, and heart) [10]. The insulin receptor (IR) consists of four subunits organized into a heterodimer: two extracellular α subunits that bind to insulin, and two transmembrane β subunits that possess tyrosine kinase activity [10]. When bound to its receptor, insulin induces its transphosphorylation on tyrosine residues, allowing its activation [11]. Activated receptors then phosphorylate several intracellular substrates, such as Src homology collagen (Shc) protein, APS (adaptor protein with PH domain and a Src homology 2 (SH2) domain) proteins, and insulin receptor substrates (IRS) [11]. 

There are six different isoforms of IRS, but only the first two are involved in insulin signal transduction. IRS1 is the isoform predominantly expressed in adipose tissue and skeletal muscle. It plays a crucial role in the propagation of the insulin signal in these tissues [12,13]. In the liver, IRS2 is expressed [12]. IRS3 exists in rodents but not in humans [14]. The IRS4 isoform is not involved in insulin signaling since its invalidation does not affect carbohydrate homeostasis in a murine model [15]. Both IRS5 and IRS6 isoforms do not transmit insulin signals [14]. Insulin-activated IR phosphorylates IRS on various specific tyrosine residues (tyrosine 612 in the case of IRS1), allowing their recognition by proteins with SH2 domains, such as Class IA phosphoinositide-3-kinase (PI3K). Activated PI3K phosphorylates phosphatidylinositol diphosphate (PIP2) to give phosphatidylinositol triphosphate (PIP3) [16], and synthesized PIP3 recruits to the PM a kinase playing a pivotal role in the transmission of the insulin signal, Akt (or protein kinase B, PKB) [17,18,19].

There are three different isoforms of Akt. Akt1 is expressed ubiquitously in cells, Akt2 is expressed predominantly in insulin-sensitive tissues, and Akt3 is mainly found in the brain [20]. Akt recruitment to the PM induces its conformational change and releases two amino acids whose phosphorylation is essential for the complete activation of the kinase. The mTORC2 (mammalian target of rapamycin (mTOR) complex 2) complex phosphorylates Akt on its serine 473, whereas phosphoinositide-dependent kinase 1 (PDK1) phosphorylates Akt on its threonine 308 site [18]. 

Akt relays the effects of insulin action in its different target tissues. In skeletal muscle and adipocytes, insulin-activated Akt induces glucose uptake via the recruitment of a specific glucose transporter, GLUT4 at the PM [21]. GLUT4 is highly expressed in skeletal muscle and adipose tissue. In the absence of insulin, GLUT4-containing vesicles are located inside the cells and less than 5% of total GLUT4 is found at the PM [22]. Upon insulin action, GLUT4-containing vesicles are recruited to the PM (30 to 50% of GLUT4 is recruited at the PM), thus allowing glucose to enter the cells [23]. In the liver and skeletal muscle, Akt phosphorylates and inhibits glycogen synthase kinase 3 (GSK3), thus allowing glycogen synthase to induce both glycogen synthesis and storage in hepatocytes and muscle cells [24]. In β-cells and the liver, the expressed glucose transporter is GLUT2, which transports glucose, fructose, and galactose, independently from insulin [25].

In healthy people, the balance between glucose production and its utilization is finely tuned. When blood glucose reaches a critical threshold level, pancreatic β-cells secrete insulin. Insulin has two major actions: to lower circulating glucose levels by facilitating its uptake, mainly into skeletal muscle, while inhibiting its production by the liver; and to promote the storage of available nutrients. Normally, the unconsumed fraction of fatty acids is stored in lipid droplets that are localized in adipocytes to provide energy during fasting periods. However, in the case of obesity, when the buffering action of adipose tissues to store fatty acids is impaired, non-adipose tissues (e.g., muscle, liver, and pancreatic β-cells) accumulate fatty acids, leading to a phenomenon called lipotoxicity. Accumulation of lipids and of their sphingolipid derivatives, such as ceramides, induces cellular toxicity and insulin signaling defects, leading to insulin resistance. Insulin resistance results in a failure of insulin-sensitive tissues to respond to the insulin signal. As soon as insulin resistance appears, the pancreas produces more insulin to maintain normoglycemia. However, when insulin secretory function is insufficient to counterbalance increasing insulin resistance, which correspond to the β-cell failure, hyperglycemia and T2D appears. This insulin secretion dysfunction is linked to genetic/epigenetic predisposition, and cellular lipotoxicity could also precipitate the relative insulin secretion deficiency.

## 3. Lipotoxicity and Muscle Insulin Resistance

During T2D, peripheral tissues lose their ability to respond to insulin. Tissue insulin resistance is multifactorial, and its starting point is still very much debated. Considering the contribution of muscle in glucose uptake in response to insulin [9], muscles play a major role in the establishment of peripheral insulin resistance. In addition, several studies have shown that muscle insulin resistance contributes significantly to the systemic alteration of glucose metabolism [26,27,28,29].

In case of a large FA supply, FA are esterified into triglycerides (TG) and stored in the adipose tissue to avoid their accumulation in the circulation. Adipose tissue can provide this buffer function with its expansion capabilities. However, when adipose tissue reaches its maximum expansion capacity and can no longer perform its storage function, circulating free FA concentrations are at least doubled in obese and T2D individuals compared with lean and healthy subjects [30], and excess lipids accumulate in other peripheral tissues, such as muscles, liver, and pancreas. In addition, low-grade inflammation is observed in adipose tissue of obese individuals [31], leading to an increase in adipocyte lipolysis, which thus contributes to an increase in plasma FA concentrations [32]. Maintenance of adipose tissue expansion is therefore very important to avoid lipid ectopic accumulation. Indeed, absence of white adipose tissue in mice induces rapid ectopic accumulation of lipids in peripheral tissues such as liver and skeletal muscle [33,34]. Interestingly, muscle fiber lipid content is twice as abundant in obese T2D patients compared to lean individuals [35,36]. In this situation, an adaptation of TG metabolism occurs. Expressions of FA transporters (fatty acid binding protein, FABP, and FA translocase, FAT/CD36) in myocyte PMs are augmented when the FA concentrations are increased in non-obese individuals on a high-fat diet for four weeks [37]. It has also been observed in obese or T2D individuals that long chain FA transport into giant sarcolemmal vesicles was four times greater than what was observed in muscle cells of healthy individuals [38].

Despite the increase in FA transport, oxidative capacities of muscle cells are non-adequate for the amounts of FA arriving in these cells. Activities of CPT1 (carnitine palmitoyltransferase 1), citrate synthase (the first enzyme in the Krebs cycle), and cytochrome c oxidase (or complex IV of the mitochondrial respiratory chain catalyzing the transfer of electrons to a molecule of O_2_, while contributing to the H^+^ proton gradient), are decreased in muscles of obese patients compared to the muscles of lean individuals [6]. In addition, muscles of obese or T2D individuals contain fewer mitochondria and are reduced in size [39,40,41], leading to a decrease in the oxidative capacities of muscle cells [42]. It has also been shown in extremely obese female individuals (with a body mass index over 53 kg/m^2^) that β-oxidation was 58% and 83% lower than muscle strips from lean and moderately obese subjects, respectively [43]. 

In addition to oxidation defects, the saturation state of FA greatly influences their fate. In fact, saturated FA are much less oxidized than unsaturated FA [44], and they accumulate much more in tissues in lipid excess conditions. Increased entry of FA is thus accompanied by a decrease of the oxidative capacities of the muscle cells, leading to lipid accumulation and the onset of insulin resistance.

### 3.1. Relationship Between FA and Insulin Resistance: Randle Cycle Hypothesis

Until 15–20 years ago, the classic “Randle cycle” hypothesis was the best explanation for understanding the reciprocal relationship between carbohydrate metabolism and lipid metabolism, and particularly the deleterious role of fatty acids on glucose metabolism in insulin-sensitive tissues.

In 1963, Randle proposed the existence of a “glucose-fatty acid” cycle that would play a crucial role in the development of cellular insulin resistance [45]. He showed that the substantial increase in intracellular free FA concentrations would result in an increase in circulating glucose and a decrease in its uptake by the cells. Randle and his team showed that the high amount of FA available in cells led to an increase in β-oxidation, inducing a massive increase in citrate content. This citrate would then inhibit phospho–fructo kinase (PFK), thereby inducing an increase in intracellular glucose-6-phosphate (G6P) and an inhibition of hexokinase, protein responsible for glucose phosphorylation into G6P. Glucose levels would therefore increase in cells, preventing more circulating glucose to be transported into cells [45].

It was not until the early 1990s that new studies put this cycle back into question. Some predictions of the Randle cycle have been jeopardized by the use of new techniques, such as nuclear magnetic resonance, which allowed measurement of metabolite concentrations in tissues in vivo. Furthermore, contrary to what Randle predicted, this technique showed that free FA accumulation induced a decrease in intracellular G6P levels [46], despite the decrease in glucose transport into cells [46]. Thus, Randle’s glucose-fatty acid cycle did not explain how FA modulate insulin action and glucose metabolism in insulin sensitive tissues.

### 3.2. Lack of Direct Link between FA and Insulin Resistance

Skeletal muscle is an important site for the clearance of lipoproteins containing TG-derived FA [39]. Most of these FA are sent to esterification in TG. Indeed, 60% of circulating FA contribute to intracellular TG [39]. Intramyocyte TG concentrations are significantly increased in obese and diabetic subjects compared with intramyocyte TG concentrations from healthy subjects [47]. However, the involvement of TG as primary initiators of the onset of insulin resistance in muscle cells is far from clear. Overexpression of the FAT/CD36 FA transporter in skeletal muscle has been shown to counteract insulin resistance induced by a fatty diet via increased myotube oxidative capacities, despite increased TG concentrations in cells [32]. 

Interestingly, there is a situation of muscle lipid accumulation that is associated with a very good insulin sensitivity. This is the “athlete paradox”. Athletes’ muscles accumulate as much intramyocyte lipids as insulin-resistant obese muscles [48]. In addition, the size of intramyocyte lipid droplets is not significantly different between both groups [48]. Thus, it does not appear that the presence of FA in the muscle cells is the direct cause of the appearance of insulin resistance of the tissue. FA are at the origin of the production of secondary lipid derivatives, such as diacylglycerols (DAG) and ceramides, and these species play a crucial role as mediators of the deleterious effects of FA on muscle insulin sensitivity.

### 3.3. Diacylglycerols

Diacylglycerols (DAG) are intermediate lipid species synthesized during the esterification of FA to TG, but also in the metabolism of phospholipids [49]. The transformation of DAG into TG involves diacylglycerol acyltransferases (DGAT) [50]. The DGAT1 isoform is expressed in all tissues but is highly expressed in adipose tissue, intestine, heart, and skeletal muscle [50]. The DGAT2 isoform is expressed in the liver and adipose tissue [50]. DAG are metabolic intermediates for the synthesis of specific phospholipids, in particular for the synthesis of phosphatidic acid (PA) [51]. This reaction is catalyzed by a family of DAG kinases (DGK), which phosphorylate DAG to give PA [51]. There are four isoforms of DAG kinases: α, δ, ε, and ζ [51]. 

DAG are known to be important second messengers for several metabolic processes in the cell, including transcription, cytoskeletal dynamics, release of neurotransmitters, and lipid signaling [52]. 

It was in hepatocytes that DAG involvement as deleterious lipids on insulin signaling was first described. In these cells, it has been demonstrated that DAG alter insulin signaling via activation of protein kinase C ε (PKCε) [53]. Indeed, once activated, PKCε phosphorylates IRS2 on serine residues, thus preventing the propagation of the insulin signal [13,53]. 

The involvement of DAG in hepatic insulin resistance is now well established and several studies show that a similar mechanism exists in muscle cells. Pioneer studies have shown that DAG concentrations were increased in muscles from obese and insulin-resistant rats [54]. Several studies showed that DAG activate PKCθ and phosphorylate IRS1 on its serine 307 residue, preventing the phosphorylation of IRS1 on its tyrosine residues in response to insulin, blocking insulin signaling, and thus inducing muscle cell insulin resistance (reviewed in [13]). 

### 3.4. Ceramides

Ceramides are known to exert a predominant structural role, but also pro-apoptotic functions, as well as an important role in cell growth arrest, differentiation, senescence, cell migration, and cell adhesion [55]. Ceramides have a structural role within the different cell membranes. In resting cells, ceramide concentrations in the PM are very low [56]. However, these concentrations may increase rapidly in response to certain stresses (e.g., cytokines, chemotherapeutic agents) [56]. In the PM, ceramides are mainly found in lipid rafts, inducing a modification of the biophysical properties of the membrane [56]. Rafts are microdomains rich in cholesterol and sphingolipids, and they allow the compartmentalization of receptors and transporters, thus facilitating or inhibiting diverse signaling pathways [57]. These modifications of membrane structures result from the very nature of ceramides, in particular their high hydrophobicity and their high melting temperature point, which induces a decrease in their miscibility with other membrane lipids [56]. Ceramides can also modify the permeability of membranes, especially the outer membrane of mitochondria, an important step in the induction of apoptosis [58], by creating channels through the mitochondrial membrane [59].

Ceramides are second major messengers of the inflammatory response and apoptosis induced by tumor necrosis factor α (TNFα) [60]. Ceramides can also activate caspases 3 and 7 and thus promote apoptosis in erythrocytes [58].

Ceramides are also involved in some cancers. However, all species of ceramides do not play the same role in proliferation or cell death of tumor cells. For example, C16-ceramides have been shown to promote proliferation of head and neck tumor cells [61]. In contrast, C18-ceramides promote tumor growth arrest, possibly through mitophagy [61]. 

Ceramides are known to induce differentiation of a hematopoietic stem cell line (HL-60) [62]. They also mimic the action of a nerve growth factor on glioma cells (T9 cell line) [62]. Ceramides also have roles in the differentiation of skin cells. Short-chain ceramides induce differentiation in human keratinocytes [63].

In addition to those roles, ceramides are now well characterized for their inhibitory action on insulin signaling.

## 4. Ceramide Metabolism and Muscle Insulin Resistance

### 4.1. Sphingolipid Biosynthesis

Ceramides, central lipids for the biosynthesis of sphingolipids (SL), are synthesized mainly via their de novo biosynthesis pathway, almost exclusively from palmitate. In addition to this pathway, ceramides can also be synthesized via the sphingomyelinase pathway and the so-called “recycling” SL pathway.

These metabolic pathways are complex and involve several reactions taking place in different cell organelles. (i) The de novo ceramide synthesis is carried out in the endoplasmic reticulum (ER). (ii) The conversion of ceramides into complex sphingolipids such as sphingomyelins and glycosphingolipids occurs mainly in the Golgi apparatus. (iii) The recycling pathway takes place both in a lysosomal compartment and the ER.

#### 4.1.1. De Novo Synthesis Pathway 

**Figure 1:**
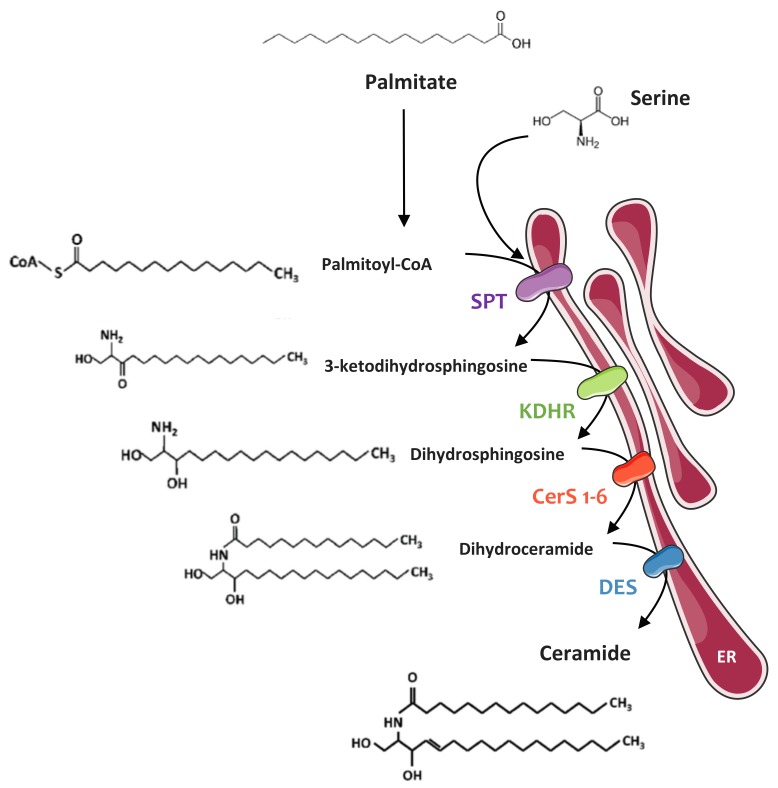
Ceramide de novo synthesis pathway. Palmitate is the preferential fatty acid used for the de novo synthesis of ceramides. The biosynthesis pathway takes place in the ER. Palmitoyl-CoA is first condensed with a serine to form a 3-ketodihydrosphingosine through the action of SPT. 3-Ketodihydrosphingosine is rapidly metabolized into dihydrosphingosine by KDHR. Formed dihydrosphingosine are acylated by different isoforms of CerS to form dihydroceramides of different chain lengths. Dihydroceramides are then desaturated by DES1 to give ceramides. ER: Endoplasmic reticulum; SPT: Serine palmitoyl transferase; KDHR: 3-ketodihydrosphingosine reductase; CerS: Ceramides synthases; DES1: Dihydroceramide Δ4-desaturase.

This pathway starts in the ER, where four enzymes (or families of enzymes) generate ceramides of different chain lengths from a non-sphingolipid precursor (palmitate) (Figure 1). This pathway is predominant in a situation of lipid excess. Ceramide synthesis begins with the condensation of l-serine with palmitoyl-CoA by serine palmitoyl-transferase (SPT), generating 3-ketosphinganine, rapidly reduced to form sphinganine via 3-ketosphinganine reductase. Sphinganine is then acylated by ceramide synthase (CerS) isoforms to form dihydroceramide. In mammals, six CerS isoforms are expressed and are called CerS 1 to 6 [58]. They carry out the same chemical reaction but display distinct specificities for the acyl-CoA chain length they use for N-acylation. Thus, CerS isoforms are responsible for the fatty acid composition of ceramides. The chain base moiety of the lipid is finally desaturated by dihydroceramide desaturase (DES1) at the 4–5 position of the sphingoid base backbone to form ceramide [64].

#### 4.1.2. Sphingomyelinase Pathway

Hydrolysis of SM is performed by sphingomyelinases (SMase), allowing the release of ceramides and phosphocholine [65]. There are three categories of SMases that are classified according to their optimal pH and their subcellular localization: acidic SMase (aSMase), neutral SMase (nSMase), and alkaline SMase. Alkaline SMase, which is only expressed in liver and intestines, plays a role in the digestion of SM from the food [65]. However, aSMase and nSMase are ubiquitously expressed and are major regulators of SM catabolism in most tissues [65]. 

#### 4.1.3. Recycling Pathway

This pathway that takes place in lysosomes is used by complex sphingolipids that are broken down into sphingosine, which is then recycled through reacylation to produce ceramide. This pathway involves a number of key enzymes that include SMases, ceramidases, and ceramide synthases [66].

### 4.2. Ceramides and Muscle Insulin Resistance

Studies carried out both in vitro and in vivo have shown that intracellular concentrations of ceramides were increased in lipotoxic situations and contributed to the development of muscle insulin resistance (Table 1).

#### 4.2.1. In Vitro Studies

A first study showed in C2C12 myotubes that palmitate-induced insulin resistance implied an increase in ceramide concentrations via their de novo biosynthesis pathway, leading to the inhibition of Akt [67], a crucial kinase from the insulin signaling pathway [18,68]. The authors cultured C2C12 myotubes in the presence of palmitate and observed a two-fold increase in ceramide concentrations in the cells. In addition, the study showed quite similar results by directly adding short-chain C2-ceramides to the cells [67]. These results were rapidly confirmed in another cellular model of myotubes, L6 myotubes, in the presence of C2-ceramides [69]. C2-ceramide treatment induced a decrease in phosphorylation of Akt on both its serine 473 and threonine 308 residues as well as a decrease in glucose uptake and glycogen synthesis in myotubes [69]. 

Another study showed that inhibition of the de novo ceramide synthesis pathway partially restored insulin sensitivity of L6 myotubes [70]. Cells were incubated with palmitate in the presence or absence of myriocin, a selective inhibitor of SPT. Myriocin prevented the palmitate-induced increase of ceramides and preserved normal activation of both Akt and glucose transport in response to insulin [70]. 

#### 4.2.2. In Vivo Studies

Many in vivo studies have confirmed the major role of ceramides in the installation of muscle insulin resistance observed in vitro (Table 1).

It has been known since the early 1990s that ceramide concentrations are increased in both soleus and plantaris muscles of obese and diabetic Zucker rats [54]. Since then, several groups have used pharmacological approaches to highlight the importance of ceramides in the development of muscle resistance in vivo.

A study conducted in mice fed a high-fat diet and treated with myriocin demonstrated that inhibition of ceramide synthesis was sufficient to partially restore insulin-stimulated Akt phosphorylation in gastrocnemius muscles [26]. This improvement in muscle Akt phosphorylation was accompanied by better glucose tolerance and insulin sensitivity [26]. 

These data were rapidly confirmed by a study showing that inhibition of ceramide synthesis could counteract glucocorticoid-induced insulin resistance [83]. Rats treated with dexamethasone, a synthetic glucocorticoid, displayed impaired glucose metabolism and insulin resistance [83]. However, treatment of these animals with myriocin restored their glucose tolerance and insulin sensitivity [83]. In the same study, authors also showed that treatment of obese Zucker rats with myriocin significantly improved their glucose tolerance and decreased their blood glucose, as a result of a decrease in plasma and tissue ceramide concentrations [83].

They also used a genetic approach to confirm the role of ceramides in insulin resistance in vivo. The authors generated DES1 knock-out (KO) mice (DES −/+). A decrease in ceramide concentrations in several tissues (heart, liver, brown adipose tissue, and soleus muscle) was observed in dexamethasone-treated mice compared to ceramide concentrations observed in wild-type mice. An increase in insulin sensitivity was observed in the DES1 KO animals compared to their wild-type littermates [83].

More recently, a study in rats fed a high-fat diet showed that myriocin treatment also helped normalizing glucose tolerance and insulin sensitivity [85]. This treatment induced a decrease in ceramide concentrations in soleus muscle [85]. Restoration of insulin-stimulated Akt phosphorylation was also observed in soleus muscle of myriocin-treated rats [85].

#### 4.2.3. Human Studies

As in obese rodent models, several studies showed that muscle ceramide concentrations were increased in obese and insulin-resistant patients compared to healthy individuals (Table 1).

A study conducted in obese and lean subjects showed a strong correlation between loss of insulin sensitivity and increased intramuscular ceramide concentrations [88]. These results suggest that high muscle concentration of ceramides of obese individuals contribute to insulin resistance.

Similar results have been described in other studies. Total ceramide concentrations were shown to be increased in vastus lateralis muscles of obese subjects compared to those of lean subjects [89]. Several other studies show results similar to those previously described [48,90,91].

Interestingly, C16- to C24-ceramide species have been shown to be the most associated with hyperinsulinemia and a strong HOMA-IR (homeostasis model assessment of insulin resistance, a test to measure the β-cell function and insulin sensitivity) [93,95].

## 5. Mechanisms of Ceramide-Mediated Muscle Insulin Resistance

Given the major role that ceramides exert on the regulation of muscle insulin sensitivity, it was legitimate to question their mode of action within cells, and ceramides have been demonstrated to target two major players in insulin signaling: Akt via two different mechanisms, and IRS1.

### 5.1. Inhibition of Akt by Ceramides

**Figure 2:**
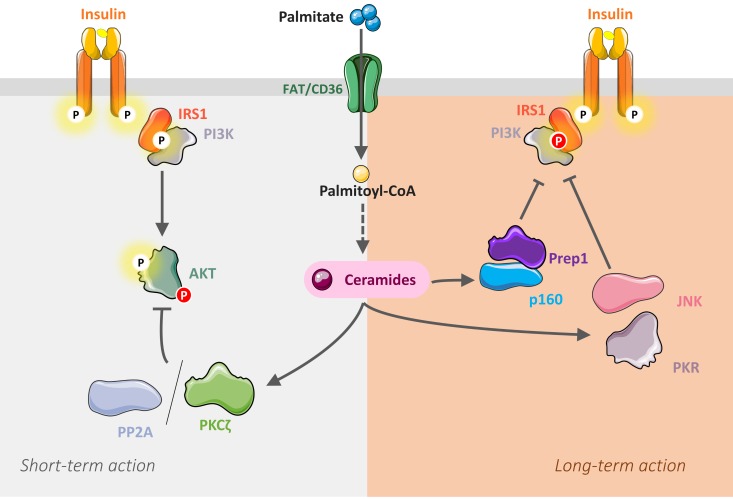
Short- and long-term action of ceramides on insulin signaling in muscle cells. Ceramides inhibit the insulin signaling pathway in muscle cells by targeting in a time-dependent manner two important actors, Akt and IRS1. Ceramides rapidly activate either PP2A or PKCζ to inactivate Akt. In the long-term, ceramides induce PKR/JNK and/or Prep1–p160 axes to target IRS1. IRS1: Insulin receptor substrate 1; JNK: c-Jun NH2-terminal kinase; PI3K: Phosphoinositide-3-kinase; PKCζ: Protein kinase C ζ; PKR: Double stranded ARN-activated protein kinase; PP2A: Protein phosphatase 2A. Prep1: Pbx regulating protein 1. Dotted arrows: indirect action; Solid arrows: direct action. P: phosphorylation.

Pioneering studies showed that the phosphatase PP2A mediated Akt inhibition by ceramides in C2C12 myotubes. When cultured in the presence of palmitate, an increase in PP2A activity and a concomitant decrease in the activity and phosphorylation of Akt in response to insulin was observed [71,73,96]. Inhibition of PP2A activity (with the PP2A inhibitor okadaic acid) completely restored insulin-induced Akt phosphorylation, thus confirming the role of PP2A in the inhibition of insulin signaling in this muscle cell model [71,73,96] (Figure 2).

A second mechanism, independent of PP2A, but implicating the atypical protein kinase C ζ (PKCζ), has been highlighted in another muscle cell line (myotubes L6) [97], in human muscle cells [78], and in adipocytes [76] (Figure 2). This second mechanism involved a loss in membrane recruitment and phosphorylation of Akt by a process dependent on activation of PKC*ζ* [72,74,75]. PKC*ζ* has been described to directly interact with and to repress the activity of Akt in several cell types [72,74,98,99,100], but to dissociate upon cell treatment with growth factors [98] and insulin [99], thereby allowing Akt to become activated. However, when intracellular ceramide contents are increased, a potent activation of PKC*ζ* is observed [69,70], stabilizing the interaction between Akt and PKC*ζ* [74]. This interaction antagonizes the ability of insulin to dissociate the kinase complex, but also to activate Akt [74] (Figure 2). Interestingly, involvement of PM microdomains called caveolae has been demonstrated in this mechanism. Caveolae are specific sites for ceramide production in response to various stresses [101]. They are small invaginations of the PM enriched in cholesterol and SL, and proteins called caveolins are very important for the formation and stability of these structures [101,102]. These caveolae are found in many cell types but are very abundant in insulin-sensitive cells such as adipocytes and muscle cells [101]. The presence of PKCζ in these caveolae, as well as its interaction with caveolins, has also been highlighted [103], and our laboratory showed that ceramides induced the recruitment and sequestration of both Akt and PKCζ in these microdomains, thus blocking the insulin signal [76]. 

Considering all these data, one question remained unresolved: why did ceramides inactivate Akt via two independent mechanisms, PP2A or PKCζ? Studies in our laboratory demonstrated that, in caveolae-poor cells (C2C12 myotubes, fibroblasts), ceramides act via PP2A on insulin signaling, whereas a PKCζ-dependent mechanism is taking place in caveolae-rich cells (adipocytes, L6 and human myotubes) [78,104].

### 5.2. Inhibition of IRS1 by Ceramides

In vivo, in rodents fed a high fat diet, skeletal muscle insulin resistance was observed usually at the level of both IRS1 and Akt [105,106,107]. However, although the mechanisms by which ceramides inhibited Akt were well demonstrated (see previous chapter), those involved a possible inhibition of IRS1 remained unclear until recently. 

Hage Hassan et al. showed that long-term incubation of C2C12 myotubes with palmitate induced IRS1 phosphorylation on serine residue, thus preventing IRS1 activation in response to insulin [79]. Similar effects were obtained by chronically incubating myotubes directly with C2-ceramides [79]. They showed that this mechanism implied the double-stranded RNA-activated protein kinase (PKR), a protein involved in the innate immune response [79]. At the time, PKR was already known to modulate negatively both IRS1 and IRS2 in a hepatic tumor cell line, and to modulate negatively insulin sensitivity in a model of obese mice fed a fat diet [108,109,110]. Hage Hassan et al. demonstrated that PKR was activated in vitro in muscle cell lines treated with palmitate or directly with ceramides, in vivo in muscles obtained from high-fat diet fed mice and from genetically obese mice, and also in human diabetic patient muscle cells [79]. They showed that long-term ceramide activation of PKR inhibited insulin-induced IRS1 activation in muscle cells in a JNK dependent manner [79]. Preventing ceramide-induced PKR or JNK activation restored muscle cell insulin sensitivity [79]. 

Interestingly, IRS1 has also been shown to be a target for ceramide-induced transcription factors Prep1 (Pbx regulating protein 1) and p160 in muscle cells [111]. Prep1 is a physiologic regulator of insulin-mediated glucose and lipid metabolism in skeletal muscle [112,113]. In muscle, Prep1 binds and stabilizes p160, thus repressing GLUT4 expression [112]. A recent study demonstrated that long-term ceramide action promoted Prep1–p160 association and impaired insulin-induced IRS1 phosphorylation in muscle cells [111]. 

Overall, in muscle cells, ceramides inhibit insulin signaling via two mechanisms: a short-term mechanism directed towards Akt through the activation of PP2A/PKCζ pathways; and longer-term mechanisms involving PKR/JNK and/or Prep1–p160 axes that target IRS1 (Figure 2).

### 5.3. Importance of Ceramide Species in the Onset of Muscle Insulin Resistance

Ceramide is not a homogeneous lipid class since several species with different chain lengths (from C14 to C26) exist. This is why more and more studies are interested in the role of these different species in the development of insulin resistance. 

In hepatocytes, for example, studies suggest that C16-ceramides play an important role in the development of insulin resistance [114,115,116] whereas C22-, C24: 1, and C24-ceramides are protective [115]. 

In muscle cells, no specific ceramide species have been confidently identified so far, but C18-ceramides have been often cited as potential candidates. Several studies have shown a positive correlation between muscle C18-ceramide content and insulin resistance [84,85,92,117]. In addition, a recent study found that C18-ceramides concentrations were increased at the level of the PM in muscle cells of T2D individuals [94]. It is important to note that these studies showed positive correlations between C18-ceramide concentrations and insulin resistance, and not causes to effect action. However, a very recent study filled this gap and highlighted a functional implication of C18-ceramide species in the pathogenesis of insulin resistance [87]. Brüning’s lab demonstrated that global or muscle specific reduction of C18-ceramide content enhanced whole-body glucose metabolism [87]. Thus, these results revealed the tissue-specificity function of C18-ceramides during the development of obesity associated insulin resistance. 

Nevertheless, a no less interesting and recent study showed conflicting data and demonstrated that the implication of C18-ceramides in the development of muscle insulin resistance was not that undisputable. Turner et al., developed a specific inhibitor of CerS1 (PO53), leading to a decrease in C18-ceramide concentrations (−50%) in the skeletal muscles of mice subjected to a high-fat diet [86]. Despite this decrease in intramuscular C18-ceramide concentrations, both the glucose tolerance and insulin sensitivity were not improved [86]. This suggests that C18-ceramides are not a main lipid actor for the development of insulin resistance in these animals or that the remaining muscle C18-ceramide content is enough to inhibit muscle insulin sensitivity. On the other hand, the authors of this paper showed that C18-ceramides played a very important role in fat storage by inhibiting mitochondrial β-oxidation [86]. Another recent study tended to lead to the same conclusion. Bandet et al. overexpressed the ceramide transporter CERT that transports newly synthetized ceramides from the endoplasmic reticulum towards the Golgi apparatus to be transformed into SM in tibialis anterior muscle from mice fed a high-fat diet [80]. Only concentrations of some ceramide species were impacted by CERT overexpression. They observed a decrease in concentrations of C16-, C22-, C24:1-, and C24-ceramide species in CERT overexpressing tibialis anterior muscles compared to control muscles, without any change in both C18- and C20-ceramide species [80]. It seems that the responsibility for insulin resistance is rather to be sought from the other ceramide species that vary in this study (C16, C22, C24:1, and C24) [80]. 

## 6. Ceramide vs DAG as Modulators of Muscle Insulin Sensitivity

Although many studies have found DAG to alter the insulin response in muscle cells, some other studies do not report such effects. A non-exhaustive list of them are presented below. 

Selathurai et al. used a mouse model in which the gene encoding to phosphoethanolamine cytidylyltransferase, an enzyme that metabolizes DAG and ethanolamine to form phosphoethanolamine, was specifically invalidated in muscles [118]. A two-fold increase in DAG concentrations of mouse muscle cells was observed without inducing insulin resistance [118]. 

Other studies show similar results. Indeed, an increase in muscle glucose uptake and insulin sensitivity was observed in mice in which muscle DAG concentrations were increased after overexpression of DGAT1 in the mouse tibialis anterior [119]. Mice fed a high-fat diet (45% fat) were treated with a β-oxidation inhibitor (etomoxir), thus leading to an intracellular accumulation of lipids, and in particular, DAG in the *anterior tibialis* anterior of the animals [120]. Insulin sensitivity was found to be improved in these muscles [120], and mice treated with the inhibitor were more glucose-tolerant than the placebo-treated mice [120].

Studies performed in humans did not report any differences in DAG concentrations in obese T2D patient myotubes compared to control subjects [47,91]. Additional studies reported identical results in obese and glucose-tolerant individuals compared to obese and glucose-intolerant individuals [90,121], and it has even been shown that muscle DAG concentrations of obese and T2D individuals were lower than those of healthy individuals [122].

Despite the important literature linking ceramides and muscle insulin resistance, there are also some studies that did not report this causal link. 

Several studies have shown that DAG are responsible for muscle insulin resistance observed in response to lipid perfusion in rats [123], in healthy human subjects [124] or in obese subjects [125], without any modification of ceramide concentrations being reported. This would suggest that ceramides did not interfere in the development of muscle insulin resistance under these particular lipotoxic conditions. It should be noted, however, that all the studies reporting a lack of ceramide effects used a lipid infusion approach, both in mice and humans, and that the lipid exposure time course remained rather short (from 1 to 9 h) [123,124,125]. One possible explanation could be that the time exposure of tissues to the lipids was not sufficient to produce enough ceramides (or at least to produce the deleterious ceramide species) since it has been shown in vitro that at least a 16 h exposure of muscle cells with palmitate was necessary to synthetize enough endogenous ceramides [67,70,73,75,126]. Therefore, it is conceivable that DAG are produced in a very short period of time (less than 9 h) and thus, they act primarily on the insulin signaling pathway, ceramides taking over later on. Further studies will surely be needed to answer these questions. 

Another study tried to demonstrate that muscle ceramide species were not implicated in the generation of insulin resistance [77]. CerS isoforms were overexpressed using adenovirus or knocked down using siRNAs in L6 muscle cells. Unexpectedly, they observed that overexpression of some CerS isoforms promoted insulin action, without any negative effect on insulin sensitivity [77]. These results were found peculiar at the time but could be explained by the very low palmitate concentrations used in the study. At that lipid concentration, no loss in insulin signal (on both IRS Akt phosphorylation), or action (on glycogen synthesis) were observed [77]. 

A very recent in vitro study from Klip’s lab also suggested that muscle ceramide accumulation did not play a causative role in the development of insulin resistance [81]. Using various inhibitors of the ceramide de novo biosynthesis pathway, they failed to see an improvement in palmitate-induced insulin resistance in L6 muscle cells [81]. These results were opposite from those obtained in the same cell line and published earlier by Powell et al. [75]. These discrepancies, however, could be explained by very important differences in palmitate response between the two studies. Pillon et al. observed only a 10 to 20% increase in intracellular ceramide content in response to palmitate [81]. In contrast, Powell et al. saw a 6-fold increase in total ceramide concentrations upon palmitate treatment in the same cells [75]. Since an increase in DAG content was also observed in these conditions [75], it is conceivable that, in the lipotoxic model used by Pillon et al. (low ceramide concentration in response to palmitate), development of insulin resistance became DAG dependent. 

DAG or ceramides are both lipids that play important roles in establishing muscle insulin resistance. As stated above, it is therefore difficult to identify which class of lipids exerts the most determining role. Nevertheless, some studies showed that ceramides have more influence than DAG on the development of muscle insulin resistance.

This has been demonstrated in an in vitro study in which L6 myotubes were cultured in the presence of both palmitate and myriocin. A restoration of insulin-stimulated Akt phosphorylation, despite an increase in DAG concentrations, was observed [70]. The dominance of ceramide influence versus DAG in muscle cells was confirmed in a recent study showing that lipin-1 silencing impaired insulin sensitivity in C2C12 myotubes [82]. Interestingly, the authors observed decreased intracellular DAG levels and increased ceramide accumulation in these cells [82]. 

Similar results were demonstrated in muscles from mice fed a high-fat diet and treated or not with myriocin. In animals treated with myriocin, muscle insulin sensitivity was improved, despite an increase in muscle DAG concentrations [26]. Comparable observations were made with equivalent protocols in rats [70,83]. 

Several studies carried out in humans showed similar results to those observed in vitro, or in animals. Ceramide concentrations were found to be increased in muscles from obese and insulin-resistant individuals compared to obese, but insulin-sensitive patients, whereas muscle DAG concentrations were unchanged [89,121]. In addition, ceramide concentrations were found to be increased in muscles of obese individuals with T2D compared to those observed in athletes highly sensitive to insulin [48,94], whereas muscle DAG concentrations were twice as large in muscles from athletic individuals compared to obese and diabetic subjects [48].

In conclusion, it seems that ceramides are the lipid species playing a determining role in the establishment of muscle insulin resistance, while the influence of DAG appears to be less crucial in this tissue.

## 7. Ceramide Lipid Derivatives and Muscle Insulin Resistance

Sphingolipid derivatives synthesized from ceramides have also been shown to modulate insulin signaling in muscle cells. 

### 7.1. Ceramide-1-phosphate

One study showed in a mouse model that the total invalidation of ceramide kinase (CERK), a kinase responsible for the formation of ceramide-1-phosphate from ceramide, protected animals from obesity and glucose intolerance induced by a fat diet [127]. In addition, CERK knockout also protected mouse adipose tissue from macrophage infiltration, thus preventing adipose tissue inflammation [127]. 

### 7.2. Sphingosine-1-phosphate

In order to give sphingosine-1-phosphate (S1P), ceramides are first deacylated by the action of ceramidases to produce sphingosine [65]. The latter can then be phosphorylated with sphingosine kinase (SphK) to form S1P [65]. There are two isoforms of SphK, SphK1, and SphK2. They catalyze the same reaction, but their subcellular localization is different. SphK1 is mainly cytosolic, whereas SphK2 is localized in the different cellular compartments, like the nucleus [128]. 

Several studies have investigated the link between S1P and insulin sensitivity. This interest is explained by the fact that S1P exerts opposite effects to those of ceramides on growth and cell survival [128]. Thus, it is conceivable that its action on insulin sensitivity could also oppose that of ceramides. However, there is no real consensus regarding the role of S1P on the regulation of insulin sensitivity. Some studies give to S1P a protective role while others report it as a contributor to insulin resistance [129]. 

### 7.3. Complex Sphingolipids: Sphingomyelin and Glucosylceramides

Ceramides are synthetized in the ER and need to be transported towards the Golgi apparatus to be metabolized into complex sphingolipids. Two types of ceramide transport exist, vesicular and non-vesicular transport [65,130].

Vesicular transport allows transport of ceramides from the ER to the cis Golgi network, independently of ATP. These ceramides will be metabolized into glucosylceramides (GlcCer). This transport has not been well described but is PI3K dependent [131]. 

The non-vesicular transport allows the trafficking of ceramides from the ER to the trans Golgi network, where ceramides are metabolized into sphingomyelin (SM). The transporter, called CERT (for ceramide transporter), works in an ATP-dependent manner [130]. CERT is part of the family of proteins containing a START (steroidogenic acute regulatory protein-related lipid transfer) domain [132]. The START domain is well conserved during evolution and is found both in plant and animal cells [132]. CERT is also known as STARD11 [132]. 

#### 7.3.1. Glucosylceramides

GlcCer are synthesized from ceramides and UDP–glucose. This reaction is catalyzed by the GlcCer synthase, localized in the cis Golgi [65]. There are hundreds of species of GlcCer [133], some being surely essential for the development of mammals since mice invalidated for the gene encoding GlcCer synthase died at the embryonic stage [65]. GlcCer serve as precursors for synthesis of gangliosides [65], after the addition of a galactose residue to give a lactosylceramide [133]. This lactosylceramide will subsequently be metabolized into monosialodihexosylganglioside (GM3) through the action of GM3 synthase [134]. GM3 will serve as a precursor for the synthesis of various other gangliosides in the cell [134]. 

GlcCer inhibit insulin signaling in C2C12 myotubes, independently of ceramides [135].

Most studies reporting effects of GlcCer on insulin sensitivity focused primarily on the action of GM3. The first study reporting an effect of this lipid showed that mice invalidated for GM3 synthase exhibited better glucose tolerance, even when they were fed a high-fat diet [134]. In addition, phosphorylation of IR was greater in GM3 synthase knockout mouse muscles [134]. 

Another study reported that inhibition of GlcCer synthesis in vivo improved blood glucose levels and insulin sensitivity in a diabetic mouse model [136]. Mechanisms that may explain the negative role of GM3 on insulin signaling have been studied in 3T3-L1 adipocytes [137] and human embryonic kidney cells (HEK 293) [138]. It has been demonstrated that, in the PM, IR was associated with caveolin 1, and that the increase in GM3 concentrations promoted the dissociation of this complex, thus inhibiting propagation of the insulin signal.

#### 7.3.2. Sphingomyelin

Once in the *trans* Golgi, ceramides are metabolized into SM through the action of sphingomyelin synthase (SMS) [65]. SM are the most abundant complex SL in mammalian cells [65]. Two SMS isoforms are expressed in cells, SMS1 and SMS2. SMS1 is localized in the Golgi apparatus, whereas SMS2 is mainly located in the PM [65]. SMS carry out the transfer of a phosphocholine group from phosphatidylcholine onto ceramides. This reaction releases a SM, but also a DAG [65].

A metabolomic study demonstrated that reduced levels of plasma C16:1-SM species were predictive of T2D [139]. Inhibition of SMS in muscle cells induced a rise in ceramide content and impaired insulin signaling [140]. In addition, obese and glucose intolerant individuals showed an increased muscle ceramide content and lower muscle SM compared to obese and normal glucose tolerant individuals [89]. Altogether, it seems that the transformation of ceramides into SM could be a way to prevent ceramides from accumulating in cells and thus to overcome their deleterious effect on insulin signaling.

A recent study performed in the lab went in that direction. Bandet et al. showed that increased concentrations of ceramides in response to saturated FA were associated with a defective transport of ceramides from the ER to the Golgi apparatus in muscle cells [80]. Overexpression of CERT in vitro in muscle cells under lipotoxic conditions or in vivo in the *anterior tibialis* muscle of mice fed a high-fat diet improved insulin sensitivity [80]. Thus, these data demonstrated that the ceramide/SM axis could open avenues to find new therapeutic targets for improvement of muscle insulin sensitivity. 

## 8. Circulating Sphingolipids

In addition to intracellular-based actions, several recent studies have also highlighted a crucial role for circulating ceramides in the development of tissue insulin resistance and T2D. It is known that plasma ceramide levels are increased in obese patients [141]. In vitro studies showed that treatment of HepG2 liver cells with palmitate was associated with an increase of extracellular ceramide, suggesting that liver cells could rapidly secrete newly synthesized ceramide in response to hyperlipidemia [142]. In agreement, it has also been shown that treatment of myotubes with LDL-containing ceramide, which are produced by hepatocytes, promoted ceramide accumulation and insulin resistance [143]. In addition to lipoproteins, it has been recently shown that extracellular vesicles, which are cell-derived membranes shed both basally and under stress conditions, could be important regulators of secreted ceramide [144]. Treatment of hepatocytes with palmitate increased the release of extracellular vesicles enriched with ceramide. Importantly, these vesicles were able to stimulate macrophage chemotaxis [144]. Recently, the group of Scherer has shown that reduction of hepatic ceramide levels by overexpressing acid ceramidase prevented hepatic steatosis, but also improved insulin action in liver and adipose tissue upon exposure to high-fat diet [145]. Conversely, overexpression of this enzyme within adipose tissue also prevented hepatic steatosis and systemic insulin resistance [145]. Even if direct muscle insulin sensitivity has not been explored in this study, these observations suggest the possible existence of inter-organ communications mediated by circulating ceramide, critical to maintaining glucose homeostasis. Therefore, more work will be required to dissect the molecular mechanisms involved in the secretion of hepatic ceramide and the action of these circulating ceramides in the development of insulin resistance.

Quantifying circulating sphingolipid concentrations has become even more important since the demonstration that some of them could be used as biomarkers to identify individuals who are at risk to develop T2D. Indeed, searching for plasma lipid species whose concentrations correlate with various parameters of glucose homeostasis and susceptibility to T2D, we recently found that levels of ceramides and their precursor dihydroceramides were closely associated with glucose intolerance and defects in insulin secretion in mice at a pre-diabetic state [146]. Interestingly, another study revealed that higher plasma ceramide content, along with saturated FA concentrations, are prospectively associated with higher fasting insulin and insulin resistance in the Strong Heart Family Study cohort (a population at high risk of diabetes) [73]. Importantly, dihydroceramides were found to be significantly elevated in the plasma of individuals from the so-called (Epidemiologic Data on the Insulin Resistance Syndrome) (DESIR) cohort who will progress to diabetes up to nine years before the onset of the disease [146]. We also found recently that circulating dihydroceramides could be good T2D biomarkers, probably better than circulating ceramides [147]. 

Altogether, these observations suggest that lowering ceramides might be a target in pre-diabetes. Therefore, these lipids may serve as early biomarkers of, and help identify, metabolic deregulation in the pathogenesis of T2D. In line with this observation, the Mayo Clinic recently introduced a diagnostic test that quantifies plasma ceramides in order to identify patients at risk of major adverse cardiac events, suggesting that circulating ceramide could become the new cholesterol [148]. 

## 9. Conclusion

Over the years, studies have demonstrated that ceramides (and some of their derivate) play a crucial and deleterious role in the regulation of tissue insulin sensitivity. In muscles, there is much evidence now to say that these lipids have a major influence on the insulin response under lipotoxic conditions. The priority, however, remains to find out for sure which ceramide species mediates palmitate-induced insulin-resistance in this tissue. Up to now, it looks like C18-ceramides could be good candidates. These efforts will be crucial to identify potential targets for the development of drugs, reducing the risk of lipid-associated disorders and their metabolic and cardiovascular diseases.

To complicate the scheme, inter-organ dialogues surely exist through the secretion of sphingolipids via lipoproteins/exosomes. We are only at the beginning of understanding how and in what circumstances circulating sphingolipids could influence (positively or negatively) the insulin response of peripheral tissues such as skeletal muscles. Clearly, more work is needed to understand how the lipid networks that exist between organs function and regulate carbohydrate homeostasis. 

## Figures and Tables

**Table 1:** Analysis of the relationship between muscle ceramide levels and insulin resistance. BMI: body mass index; C2-cer: C2-ceramides; C6-cer: C6-ceramides; Cer: ceramides; DES1: dihydroceramide desaturase 1; NGT: normal glucose tolerant; IGT: Impaired glucose tolerant; HFD: high fat diet; Pal: palmitate; SPT: serine palmitoyl transferase; T2D: type 2 diabetes; [ ]: concentration; ND: not described.

First Author (Year)	Ref.	Model (Vitro)	Intervention	Ceramide Content	Change in Insulin Resistance
Schmitz-Peiffer (1999)	[67]	C2C12 myotubes	Pal / C2-cer	↗ [total Cer]	↗
Hajduch (2001)	[69]	L6 myotubes	C2-cer	ND	↗
Cazzolli (2001)	[71]	C2C12 myotubes	Pal	ND	↗
			Pal + PP2A inhibition		↘
Bourbon (2002)	[72]	Smooth muscles cells (a7r5)	C2-cer / C6-cer	ND	↗
			C2-cer / C6-cer + PKCζ inhibition	ND	↘
Chavez (2003)	[73]	C2C12 myotubes	Pal	↗ [long chain Cer, total Cer]	↗
Powell (2003)	[74]	L6 myotubes	C2-cer	ND	↗
			C2-cer + PKCζ inhibition	ND	↘
Powell (2004)	[75]	L6 myotubes	Pal / C2-cer	↗ [total Cer]	↗
			Pal / C2-cer + SPT inhibition	↘ [total Cer]	↘
			Pal/C2-cer + PP2A/PKCζ inhibition	ND	↘
Hajduch (2008)	[76]	L6 myotubes	C2-cer	ND	↗
			C2-cer + PKCζ inhibition	ND	↘
Watson (2009)	[70]	L6 myotubes	Pal	↗ [total Cer]	↗
			Pal + SPT inhibition	↘ [total Cer]	↘
Frangioudakis (2013)	[77]	L6 myotubes	Pal + CerS (1,2,4,5,6) overexpression	↗ [some species] depending on CerS overexpressed	↘
			Pal + CerS(1,2,4,5,6) knockdown	↘ [some species] depending on CerS Knockdowned	No effect on insulin signaling
Mahfouz (2014)	[78]	C2C12 myotubes / L6 myotubes	Pal / C2-cer	ND	↗
			Pal / C2-cer + PP2A/PKCζ inhibition	ND	↘
		Human myotubes	Pal	↗ [C16:0, C18:0, C20:0]	↗
			Pal + SPT inhibition	↘ [C16:0, C18:0, C20:0]	↘
Hage Hassan (2016)	[79]	C2C12 myotubes	Pal / C2-cer	↗ [total Cer] when Pal / ND when C2-cer	↗
			Pal / C2-cer + SPT inhibition	↘ [total Cer] when Pal / ND when C2-cer	↘
		Human myotubes	Pal	ND	↗
			Pal + PKR inhibition	ND	↘
Bandet (2018)	[80]	C2C12	Pal	↗ [total Cer]	↗
			Pal + SPT inhibition	↘ [total Cer]	↘
Pillon (2018)	[81]	L6 myotubes	Pal	↗ [total Cer]	↗
			Pal + SPT / CerS inhibition	↘ [total Cer]	No effect on insulin signaling
Huang (2016)	[82]	C2C12 myotubes	Lipin-1 inhibition	↗ [C16:0, C22:0, C24:0]	↗
**First author (year)**	**Ref.**	**Model (rodents)**	**Intervention**	**Muscle ceramide content**	**Change in insulin resistance**
Turinsky (1990)	[54]	Zucker rats	/	↗ [total Cer]	↗
Holland (2007)	[83]	Mice	DES1 haploinsufficiency	↘ [total Cer]	↘
		Rats	Dexamethasone + SPT inhibition	ND	↘
			Lipids infusion + SPT inhibition	↘ [total Cer]	↘
		Zucker rats	SPT inhibition	↘ [total Cer]	↘
Ussher (2010)	[26]	Mice	HFD	↗ [total Cer]	↗
			HFD + SPT inhibition	↘ [total Cer]	↘
Turner (2013)	[84]	Mice	HFD	3weeks: ↗ [C18:0]; 16weeks: ↗ [C16:0, C18:0]	↗
Blachnio-Zabielska (2016)	[85]	Rats	HFD	↗ [C14:0, C18:0, C18:1, C24:1, C24:0, total Cer]	↗
			HFD + SPT inhibition	↘ [C16:0, C18:0, C18:1, C20:0, total Cer]	↘
Hage Hassan (2016)	[79]	Mice	HFD	ND	↗
			HFD + SPT inhibition	ND	↘
Turner (2018)	[86]	Mice	HFD + CerS1 inhibition	↘ [C18:0, C18:1]; ↗ [C22:0, C24:0, C24:1, total Cer]	↗
Bandet (2018)	[80]	Mice	HFD	↗ [total Cer]	↗
			HFD + CERT overexpression	↘ [C16:0, C22:0, C24:0, C24:1]	↘
Turpin-Nolan (2019)	[87]	Mice	HFD	↗ [C14:0, C18:0]; ↘ [C26:0]	↗
			HFD + CerS1 KO	↗ [C16:0, C22:0, C22:1, C24:0, C24:1] ↘ [C18:0]	↘
			HFD + CerS1 KO muscle specific	↗ [C22:1, C24:0, C24:1]; ↘ [C18:0, C18:1, C22:0]	↘
**First author (year)**	**Ref.**	**Model (*human*)**	**Intervention**	**Muscle ceramide content**	**Change in insulin resistance**
Adams (2004)	[88]	Lean (*n* = 10) and obese (*n* = 10)	/	↗ [C16:0, C18:0, C20:0, C22:0, C24:0, C24:1, total Cer] compared to lean	↗ (total and in muscle) in obese compared to lean
Straczkowski (2007)	[89]	Lean (*n* = 12), NGT (*n* = 12) or IGT (*n* = 9) obese, healthy offspring of T2D people (*n* = 12)	/	↗ [total Cer] in offspring and IGT obese compared to lean; ↗ [total Cer] in ITG obese compared to others	ND
Coen (2010)	[90]	Women obese insulin resistant (*n* = 12) or insulin sensitive (*n* = 10)	/	↗ [C14:0, C16:0, C18:0, total Cer, saturated Cer, unsaturated Cer]	↗ in insulin resistant obese compared to insulin sensitive obese
Amati (2011)	[48]	Lean (*n* = 7), athletes (*n* = 14), IGT obese (*n* = 21)	/	↗ [C18:1, C24:0, C24:1, total]; ↘ [C14:0]	↗ in obese compared to others; ↘ in athletes compared to others
Coen (2013)	[91]	Women lean (*n* = 8) or obese (2 groups: 30<BMI<34,9 (*n* = 7) and BMI > 35 (*n* = 15))	/	↗ [C14:0, C20:1, C22:1, C24:0, C24:1] in the two groups of obese	↗ in obese (30 < BMI < 34.9) compared to lean; ↗ in obese (BMI > 35) to others
Bergman (2016)	[92]	Obeses (*n* = 14) / T2D (*n* = 15) / athletes (*n* = 15)	/	↗ [C18:0] in T2D vs obese and athletes; ↗ [C24:0] in athletes vs obese and T2D	↘ in muscle of athletes compared to others;
Broskey (2018)	[93]	Obese without T2D (*n* = 62) and obese with T2D (*n* = 44)	/	↗ [C18:1, C20:0, C22:0, C24:0, C24:1 total Cer]	↗ in obese with T2D compared to obese without T2D
Perreault (2018)	[94]	Lean (*n* = 15) / athletes (*n* = 16) / obese without T2D (*n* = 15) / obese with T2D (*n* = 12)	/	↗ [Cer total] in total muscle of T2D compared to others; ↗ [C16:0, C18:0, Cer total] in sarcolemma of T2D compared to others; ↗ [C18:0, Cer total] in nucleus of T2D compared to others	↗ in T2D compared to others; ↗ in obese compared to lean and athletes

## Abbreviations

aSMase	Acid sphingomyelinase
C1P	Ceramide-1-phosphate
CerS	Ceramide synthase
CERT	Ceramide transporter
DAG	Diacylglycerols
DES	Dihydroceramide Δ4-desaturase
FA	Fatty acids
FABP	Fatty acid binging protein
FAT	Fatty acid translocase
FATP	Fatty acid transport protein
G6P	Glucose-6-phosphate
GlcCer	Glucosylceramides
GLUT4	Glucose transporter 4
GM3	Monosialodihexosylganglioside
GM3S	GM3 synthase
GS	Glycogen synthase
SK3	Glycogen synthase kinase 3
IR	Insulin receptor
IRS	Insulin receptor substrate
JNK	c-Jun NH2-terminal kinase
KDHR	3-Ketodihydrosphingosine reductase
nSMase	Neutral sphingomyelinase
PC	Phosphatidylcholine
PE	Phosphatidylethanolamine
PFK	Phospho-fructo-kinase
PH	Pleckstrin homology
PI3K	Phosphoinositide-3-kinase
PKR	Double stranded ARN-activated protein kinase
PM	Plasma membrane
PP2A	Protein phosphatase 2A
ER	Endoplasmic reticulum
S1P	Sphingosine-1-phosphate
SL	Sphingolipids
SM	Sphingomyelin
MS	Sphingomyelin synthase
SphK	Sphingosine kinase
SPT	Serine palmitoyl transferase
T1D	Type 1 diabetes
T2D	Type 2 diabetes
TG	Triglyceride
TNFα	Tumor Necrosis Factor α
